# Diversity of resistant determinants, virulence factors, and mobile genetic elements in *Acinetobacter baumannii* from India: A comprehensive in silico genome analysis

**DOI:** 10.3389/fcimb.2022.997897

**Published:** 2022-11-28

**Authors:** Shital N. Kumkar, Ekta E. Kamble, Nikeeta S. Chavan, Dhiraj P. Dhotre, Karishma R. Pardesi

**Affiliations:** ^1^ Department of Microbiology, Savitribai Phule Pune University, Pune, Maharashtra State, India; ^2^ National Centre for Cell Science, Savitribai Phule Pune University Pune, Maharashtra State, India

**Keywords:** antibiotic resistance genes (ARGs), multidrug resistance (MDR), virulence factor genes (VFGs), mobile genetic elements (MGEs), India, *Acinetobacter baumannii*

## Abstract

**Introduction:**

The frequency of infections associated with multidrug resistant *A. baumannii* has risen substantially in India. The use of next-generation sequencing (NGS) techniques combined with comparative genomics has great potential for tracking, monitoring, and ultimately controlling the spread of this troublesome pathogen. Here, we investigated the whole genome sequences of 47 A*. baumannii* from India.

**Methods:**

In brief, *A. baumannii* genomes were analyzed for the presence of antibiotic resistance genes (ARGs), virulence factors genes (VFGs), and mobile genetic elements (MGEs) using various in silico tools. The AbaR-type resistance islands (AbaRIs) were detected by examining the genetic environment of the chromosomal *comM* gene. Multilocus sequence types were determined using the Pasteur scheme. The eBURST and whole genome SNPs-based phylogenetic analysis were performed to analyze genetic diversity between *A. baumannii* genomes.

**Results and discussion:**

A larger number of *A. baumannii* isolates belonging to the ST2 genotype was observed. The SNPs-based phylogenetic analysis showed a diversity between compared genomes. The predicted resistome showed the presence of intrinsic and acquired ARGs. The presence of plasmids, insertion sequences, and resistance islands carrying putative ARGs conferring resistance to antibiotics, quaternary ammonium compounds, and heavy metals was predicted in 43 (91%) genomes. The presence of putative VFGs related to adherence, biofilm formation and iron uptake was observed in the study. Overall, the comprehensive genome analysis in this study provides an essential insight into the resistome, virulome and mobilome of *A. baumannii* isolates from India.

## Introduction

The increasing spread of hospital-associated multidrug resistant (MDR) *Acinetobacter baumannii* strains is a health concern at the global level. The emergence of *A. baumannii* as a successful nosocomial pathogen is generally attributed to three aspects; firstly, its capacity to exhibit multidrug resistance through more than one type of mechanism. Examples include, enzymatic degradation of antibiotics, modification of target sites, upregulation of efflux pumps, and permeability alterations in the outer membrane (Asif et al., 2018; Mulani et al., 2019). The expression of multiple virulence factors (VFs) is the second attribute. Biofilm-associated proteins, lipopolysaccharides, capsular polysaccharides, porins, pili, phospholipase, outer membrane vesicle, iron, zinc acquisition system, and protein secretion systems are examples of essential VFs that assist *A. baumannii* in exhibiting antibiotic resistance, environmental persistence, host-pathogen interactions, and immune evasion (Sahu et al., 2012a; Sahu et al., 2012b; Gaidhani et al., 2014; Harding et al., 2018; Moubareck and Halat, 2020). The third critical aspect that makes *A. baumannii* a formidable pathogen is its propensity to readily acquire and spread resistance genes through different mobile genetic elements (MGEs) such as plasmids, insertion sequences, transposons, and resistance islands (Pagano et al., 2016; Cerezales et al., 2020).

In recent years, the prevalence of *A. baumannii* inflicted nosocomial infections in Indian health care systems is observed (Banerjee et al., 2018; Kumari et al., 2019). The reduced susceptibility of this pathogen to multiple antibiotics poses a therapeutic and economic burden (Asim et al., 2016). The reports also highlight a significant relationship between MDR *A. baumannii* and a higher mortality rate (Gandra et al., 2019; John et al., 2020). In the last two decades, many studies giving valuable insight into the resistance profile and prevalence of *A. baumannii* from different regions of India have been reported (Sengupta et al., 2001; Prashanth and Badrinath, 2006; Yavankar et al., 2007; Sivaranjani et al., 2013; Banerjee et al., 2018). The investigations exploring the epidemiology and endemicity of this nosocomial pathogen have also been carried out (Mathai et al., 2012; Dash et al., 2013). With the advancement in molecular techniques, many PCR- based oligotyping studies from India have effectively detected different types of intrinsic or acquired resistance determinants in clinically significant *A. baumannii* (Jones et al., 2014; Rynga et al., 2015; Pragasam et al., 2016; Kumar et al., 2019). Furthermore, several studies have used a combination of phenotypic and/or molecular techniques to establish a linkage between the presence of plasmids, insertion sequences, integron cassettes, and AbaRI in the carriage of antibiotic resistance in *A. baumannii* (Pardesi et al., 2007; Khajuria et al., 2014; Saranathan et al., 2014; Jose et al., 2017). Nevertheless, although most of the aforementioned studies are informative, they deal with a limited number of ARGs, VFGs or MGEs.

Contrary to these numerous individual phenotypic or genotypic characterization reports, whole genome sequencing can offer an overall insight into the architecture of *A. baumannii*. The prior *A. baumannii* genome sequences from India gave a summarized account of resistance genes present in the genomes (Singh et al., 2013; Kumar et al., 2015; Mahalingam et al., 2016). Apart from these individual genome studies, few other investigators attempted to compare multiple *A. baumannii* genomes from India, however the number of such studies remained limited (Balaji et al., 2015; Vijayakumar et al., 2018). Later, in 2019 a notable comparative genome analysis explained in-depth account of niche specific genome expansion by comparing complete genome of *A. baumannii* DS002, a soil isolate against 78 other *A. baumannii* publicly available genomes (Yakkala et al., 2019).

The future of pathogenic infection management in the impending post-antibiotic era depends upon the application of advanced genomics combined with functional analysis. Considering this grave scenario, the use of high-throughput sequencing techniques to investigate the outbreak, transmission, resistome, virulome, and mobilome of MDR *A. baumannii* isolates has become a need of the hour. This study aimed to determine diversity amongst the putative genetic determinants associated with antimicrobial resistance, virulence, and hyper genome plasticity in clinically significant *A. baumannii* isolates circulating in India. The present study provides a comparative genome analysis of 47 A*. baumannii* isolates collected from India during 2005-2020.

## Materials and methods

### Study design

We have performed a genome-wide comparison of 47 A*. baumannii* isolates reported from India. We screened the microbial database at NCBI for the complete genome sequences of *A. baumannii* isolates collected from Indian hospitals. We found a total of 278 A*. baumannii* genomes out of which 42 were complete. These genomes belonged to isolates reported from seven different hospitals across India during 2014-2020. In addition, five *A. baumannii* isolates, namely, AIIMS5, AIIMS7, MMC18, SRMC9, and SRMC18, collected in 2005 and maintained as a part of our laboratory collection were also sequenced in this study. The general information regarding the collection year, specimen type, and geographical location of all *A. baumannii* genomes involved in this study is given in **Table 1**.

**Table 1:** The general details and genomic features of 47 Indian *A. baumannii* analyzed in the study.

*A. baumannii* isolates	Specimen	Collection year	Hospital #	Accession number	Genome size (Mb)	GC %	CDS	tRNAs	rRNAs	ST^Pas^	ARGs	VFGs	ISs	Tns	Prophages	AbaRI size (bp)
AIIMS5*	Blood	2005	AIIMS ND	JAKZFE000000000	3.86	38.92	3629	60	3	2	24	50	2	1	3	21973
AIIMS7*	Blood	2005	AIIMS ND	JAKZFF000000000	3.94	39.01	3607	55	3	1	22	48	11	1	6	17748
MMC18*	Wound	2005	MMC TN	JAKZFG000000000	4.03	38.84	3782	61	3	494	18	43	13	Ab	6	Ab
SRMC9*	Urine	2005	SRMC TN	JAKZFH000000000	3.84	38.99	3642	61	3	150	16	52	7	Ab	4	Ab
SRMC18*	Wound	2005	SRMC TN	JAKZFI000000000	3.96	38.98	3600	59	3	94	19	50	17	Ab	2	Ab
B8342	Blood	2014	CMC TN	GCA_001077555.2	3.95	39.06	3798	74	18	1545	16	46	34	Ab	8	Ab
B8300	Blood	2015	CMC TN	GCA_001077965.2	3.85	39.25	3718	73	18	1549	16	47	35	Ab	6	Ab
VB23193	Blood	2015	CMC TN	GCA_002762545.2	4.23	39.08	4278	75	18	2	34	50	16	3	8	90431
VB31459	Blood	2017	CMC TN	GCA_003627485.2	4.15	39.08	4242	73	18	2	37	50	38	2	7	Ab
ACN21	Blood	2018	SGRH ND	GCA_004768705.1	4.05	38.96	3932	73	18	85	23	48	79	1	8	13491
CIAT758	Blood	2018	TMC WB	GCA_004758865.1	4.17	39.04	4184	74	18	10	24	41	66	1	12	18303
P7774	Pus	2018	PGIMER CD	GCA_005518095.1	4.37	39.02	4263	73	18	25	29	49	46	2	6	18301
VB33071	Blood	2018	CMC TN	GCA_005280695.1	4.01	38.98	3869	56	18	2	34	50	20	2	4	19123
VB35179	Blood	2018	CMC TN	GCA_005280395.1	4.37	39.11	4536	73	18	1512	31	50	56	3	7	18307
VB35435	Blood	2018	CMC TN	GCA_005280415.1	4.06	38.93	4132	73	18	575	21	42	35	1	8	18299
VB35575	Blood	2018	CMC TN	GCA_005280715.1	4.04	39.13	3909	59	18	2	38	51	26	2	4	66198
6507	NM	2019	ICMR ND	GCA_009455505.1	4.09	39.04	3956	74	18	2	35	50	14	1	6	19123
PM2098	Blood	2019	AIIMS ND	GCA_010500455.1	4.02	38.91	3998	49	3	10	33	42	43	2	6	Ab
PM192696	Sputum	2019	AIIMS ND	GCA_012935065.1	4.05	38.91	3883	73	18	2	30	50	16	1	4	52776
PM193665	Sputum	2019	AIIMS ND	GCA_012935085.1	4.18	39.04	4072	73	18	10	32	42	49	2	8	18305
PM194188	Sputum	2019	AIIMS ND	GCA_012935105.1	4.19	39.03	4088	73	19	10	32	41	49	2	7	18305
PM194229	Sputum	2019	AIIMS ND	GCA_012935125.1	4.13	39.07	3981	73	18	10	28	42	47	2	6	Ab
PM1912235	Sputum	2019	AIIMS ND	GCA_012935045.1	3.94	39.03	3721	72	18	2	31	50	15	3	4	23937
SP304	Sputum	2019	CMC TN	GCA_005280675.1	3.8	38.88	3672	73	18	285	15	42	8	Ab	3	Ab
VB82	Blood	2019	CMC TN	GCA_012934905.1	4.43	39.09	4300	73	18	25	28	49	26	2	9	18305
VB473	Sputum	2019	CMC TN	GCA_012934925.1	3.96	39.06	3745	73	18	2	30	51	28	2	2	23937
VB723	Blood	2019	CMC TN	GCA_012934945.1	3.93	39.07	3750	74	18	2	31	50	15	3	4	52776
VB958	Blood	2019	CMC TN	GCA_005280355.1	4.21	38.98	4278	76	23	2	31	51	61	2	6	23932
VB1190	Blood	2019	CMC TN	GCA_005280375.1	3.97	39.04	3869	70	18	2	30	51	35	1	4	23925
VB2107	Sputum	2019	CMC TN	GCA_012935185.1	4.02	39.04	3897	73	18	2	32	50	15	3	7	49926
VB2139	Sputum	2019	CMC TN	GCA_012935165.1	4.02	39.98	3841	66	19	2	30	51	15	1	3	53951
VB2181	Sputum	2019	CMC TN	GCA_012934985.1	3.95	39.1	3776	73	18	2	31	50	14	3	7	23937
VB2200	Sputum	2019	CMC TN	GCA_012935025.1	4.02	38.97	3839	74	18	2	30	51	15	1	3	53951
VB2486	Sputum	2019	CMC TN	GCA_012935005.1	4.28	39.18	4148	74	18	1	27	48	46	2	12	14136
VB7036	Blood	2019	CMC TN	GCA_012935145.1	4.06	39.12	3902	72	19	2	31	50	18	1	6	23937
VB11737	Blood	2019	CMC TN	GCA_012934965.1	4.07	38.89	3912	73	18	2	30	50	19	1	4	Ab
VB16141	Blood	2019	CMC TN	GCA_005280435.1	4.28	39.08	4153	74	18	622	28	48	44	2	6	18302
KSK sensitive	Sputum	2020	JNU ND	GCA_017726575.1	3.9	39.02	3709	74	18	374	17	47	19	Ab	4	Ab
KSK1	Sputum	2020	JNU ND	GCA_017639875.1	4.36	38.96	4202	72	15	622	28	49	59	2	6	Ab
KSK2	Sputum	2020	JNU ND	GCA_017742855.1	4.4	38.97	4245	74	18	622	29	49	59	2	6	Ab
KSK6	Sputum	2020	JNU ND	GCA_017723975.1	4.39	38.97	4232	74	18	622	29	49	57	2	6	18305
KSK7	Sputum	2020	JNU ND	GCA_017724115.1	4.4	38.97	4235	74	18	622	29	49	57	2	6	18305
KSK10	Sputum	2020	JNU ND	GCA_017724155.1	4.42	38.96	4265	74	18	622	29	49	59	2	6	18305
KSK11	Sputum	2020	JNU ND	GCA_017724195.1	4.39	38.97	4242	74	18	622	29	49	59	2	6	Ab
KSK18	Sputum	2020	JNU ND	GCA_017724215.1	4.39	38.97	4237	74	18	622	29	49	57	2	6	18305
KSK19	Sputum	2020	JNU ND	GCA_017726495.1	4.41	38.96	4257	74	18	622	29	49	59	2	6	18305
KSK20	Sputum	2020	JNU ND	GCA_017726555.1	4.39	38.97	4241	74	18	622	29	49	57	2	6	18305

‘*’denotes the genomes sequenced in this study.

‘#’denotes hospitals from where the isolates were collected.

AIIMS ND, All India Institute of Medical Sciences, New Delhi; MMC TN, Madras Medical College, Tamil Nadu; SRMC TN, Sri Ramchandra Medical College, Tamil Nadu; CMC TN, Christian Medical College Tamil Nadu; SGRH ND, Sir Ganga Ram hospital New Delhi; TMC WB, Tata Medical Centre West Bengal; PGIMER CD, Postgraduate Institute of Medical Education and Research, Chandigarh; ICMR ND, Indian Council of Medical Research, New Delhi; JNU ND, Jawaharlal Nehru University, New Delhi; NM, Not mentioned; Ab, Absent; CDS, coding DNA sequences; ST, sequence type; Pas, Pasteur; ARGs, antibiotic resistance genes; VFGs, virulence factor genes; ISs, insertion sequences; Tns, Transposons; AbaRI, *A. baumannii* associated resistant island.

### Genome sequencing, assembly, and annotations

The genomic DNA from five *A. baumannii* isolates was extracted using the NaCl-CTAB method previously described (Wilson, 2001). The quality and quantity of the isolated DNA were checked on DeNovix DS-11 Spectrophotometer (DeNovix, Wilmington, DE). The QC passed DNA was used to prepare paired-end DNA libraries using the Illumina Nextera XT kit (Illumina, San Diego, CA) following the manufacturer’s guidelines. The DNA libraries were sequenced using Illumina NextSeq 500 platform with 2 x150 bp chemistry. The NGS QC toolkit v2.3.3 was used for quality check and filtering of raw reads using default parameters (PHRED quality score - 20) (Patel and Jain, 2012). Filtered high-quality reads were used to generate draft-genome using SPAdes *de-novo* assembler v3.11.1 (Bankevich et al., 2012). The genome coverage was calculated using BBMap v38.47 (https://sourceforge.net/projects/bbmap). The quality of assembled genome was checked using QUAST v5.0.2 (Gurevich et al., 2013). The annotations of all 47 A*. baumannii* genomes were carried out using Prokka v2.1.1 and comprehensive genome analysis service provided at Pathosystems Resource Integration Center (PATRIC) v3.6.5 (Seemann, 2014; Wattam et al., 2016).

### Prediction of antibiotic resistance genes (ARGs)

The prediction of putative ARGs belonging to different resistance mechanisms was carried out using the Resistance Gene Identifier (RGI) tool provided at the Comprehensive Antibiotic Resistance Database (CARD) (Mcarthur et al., 2013). To identify ARGs, the *A. baumannii* genome assemblies were queried against the CARD database using perfect and strict criteria. The predicted ARGs showing sequence similarity between 97% to 100% were further classified into different resistance mechanisms (Alcock et al., 2020).

### Prediction of virulence factor genes (VFGs)

The putative VFGs present in *A. baumannii* genomes were predicted using an automatic pipeline called VFanalyzer provided at http://www.mgc.ac.cn/cgi-bin/VFs/v5/main.cgi (Liu et al., 2018). The VFanalyzer conducts a thorough search of the sequence similarity of VFGs with the known and predicted VFGs available in the virulence factors database for bacterial pathogens (VFDB). The identified VFGs were further classified into nine VF categories.

### Prediction of mobile genetic elements (MGEs)

The prediction of plasmids in AIIMS5, AIIMS7, MMC18, SRMC9, and, SRMC18 genomes sequenced in this study was carried out using PlasmidSPAdes (Antipov et al., 2016). For analysis, the raw reads were used and based on the median chromosomal coverage the plasmid contigs were filtered. The filtered sequences were annotated using PATRIC v3.6.5. The plasmid sequences of 42 other *A. baumannii* genomes available at NCBI were downloaded. The presence of ARGs in all plasmid sequences was detected using CARD. The presence of insertion sequences and transposons was detected through a manual BLAST analysis of each *A. baumannii* genome against the ISfinder database (Siguier et al., 2006). The insertion sequences and transposons with an e-value equal to 0.0 were selected and further evaluated for the presence of ARGs. The prophage regions present within genome sequences were detected using the PHAge search tool enhanced release (PHASTER) database (Arndt et al., 2019). The putative prophage regions were further classified into bacteriophage families. The detection of AbaRIs was manually done by observing the genetic environments of the chromosomal comM gene using the interactive genome browser provided at the PATRIC server. The NCBI BLAST was used to align detected resistance island sequences against the sequences of previously known resistance islands (Altschul et al., 1990). The earlier proposed naming system for AbaRIs was used to classify the AbaRI variants predicted in this study (Bi et al., 2019). The annotations of detected AbaRIs regions obtained using PATRIC genome browser were used to construct AbaRIs gene maps in R 4.5.0.

### Multi-locus sequence typing (MLST) and eBURST analysis

The web-based sequence typing of the 47 A*. baumannii* genomes was carried out using MLST v2.0 services available at http://www.genomicepidemiology.org (Larsen et al., 2012). For the sequence typing, we used the Pasteur scheme which is designed to identify the seven house-keeping genes namely 60-kDa chaperonin (*cpn60*), elongation factor EF-G (*fusA*), citrate synthase (*gltA*), CTP synthase (pyrG), homologous recombination factor (*recA*), 50S ribosomal protein L2 (*rplB*), and RNA polymerase subunit B (*rpoB*). Furthermore, to understand the possible genetic relatedness between ST profiles we performed eBURST analysis using the goeBURST algorithm available at http://goeBURST.phyloviz.net (Francisco et al., 2009). To determine founding STs, we uploaded the numeric data of ST profiles obtained from MLST 2.0 in the flat text file format to goeBURST v1.2.1.

### Phylogenetic analysis

The phylogenetic relationship between 47 A*. baumannii* genomes was determined through multiple sequence alignment of genomes based on the single nucleotide polymorphisms (SNPs). The genome alignment and detection of SNPs in each genome were carried out using novel SNP procedure provided at http://cge.cbs.dtu.dk/services/CSIPhylogeny/ (Kaas et al., 2014). The novel SNP procedure filters, concatenates, aligns, and constructs a maximum likelihood phylogenetic tree with 100 bootstraps. The *A. baumannii* ATCC 19606 genome was used as a reference. The final version of the phylogenetic tree was visualized in iTOL v6 (Letunic and Bork, 2021).

### Statistical analysis

The Welch’s t-test was performed to identify difference between the number of acquired ARGs predicted between genomes (n_1_ = 18, ${\bar{\text{x}}}_{2}=8.83$ , S_1_ = 2.43, ${\text{S}}_{1}^{2}=5.91$ ) belonging to ST2 genotype and genomes (n_2_ = 29, ${\bar{\text{x}}}_{2}=7.83$ , S_2_ = 5.21, ${\text{S}}_{2}^{2}=28.15$ ) belonging to other STs detected in the study. The difference was considered significant at p<0.05.

## Results

### Genome sequencing and assembly statistics

The assembled genomes of AIIMS5, AIIMS7, MMC18, SRMC9, and, SRMC18 showed an average of 38.95% GC content. The average assembly size of five sequenced genomes ranged between 3.7 to 3.8 Mb. The comprehensive genome analysis using PATRIC3.6.5 predicted a mean total of 3652 protein-coding sequences, 276 subsystems, and 62 RNAs. The **Supplementary Table S1** represents the assembly statistics for five *A. baumannii* genomes. The **Table 1** provides a summarized account of genomic features of all 47 Indian *A. baumannii* genomes analyzed in this study.

### MLST and eBURST analysis

The web-based MLST analysis of 47 genomes using the Pasteur scheme predicted a total of 15 STs. Out of the 47 genomes analyzed, 18 (38.3%) belonged to ST2, while 10 (21.2%) belonged to ST622. We also detected presence of ST10 (n=5), ST25 (n=2), and ST1 (n=2) in the study. The remaining ten isolates belonged to the ten STs namely, ST85, ST94, ST150, ST285, ST374, ST494, ST575, ST1512, ST1545, and ST1549.

**Figure 1:**
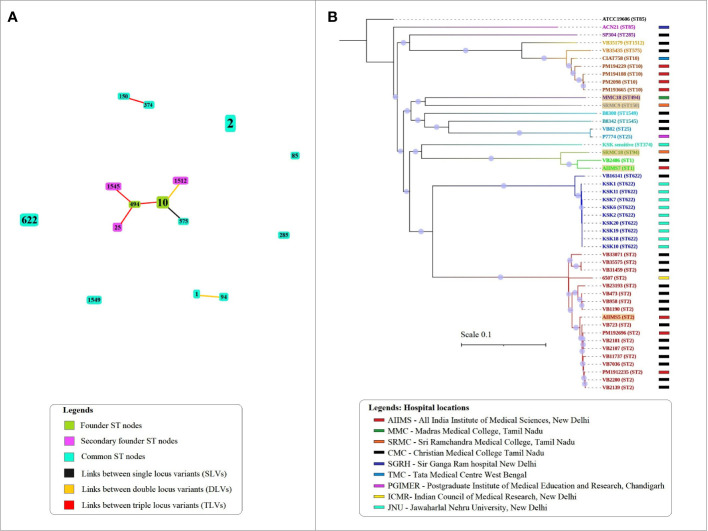
The sequence types and phylogenetic relationship between *A. baumannii* isolates analyzed in the study. **(A)** The eBURST analysis showing relationship between 15 STs detected in the population using the Pasteur scheme. The size of nodes is proportional to the number of isolates presenting that ST in the population under study. **(B)** The alignments of concatenated SNPs with 100 bootstrap replicates explain the phylogenetic relationship between 47 Indian *A. baumannii* genomes. The *A. baumannii* ATCC 19606 was used as the reference genome for alignment and SNP calling. The tree is rooted at reference genome. Different colors represent the grouping by sequence types.

The eBURST analysis grouped STs into clusters based on single, double, and triple locus variation between ST profiles detected. A total of three clusters and five singletons were observed in the population of 15 STs (**Figure 1A**). The largest cluster included six STs namely, ST10, 494, 575, 1512, 1545, and 25. In this cluster, ST10 and ST575 were single locus variant while ST10 and ST1512 were double locus variants. The remaining STs in this cluster were triple variants. The second cluster included the double locus variant ST1 and ST94 while the third cluster included triple locus variants ST150 and ST374.

### SNP-based phylogenetic analysis

The SNP-based phylogeny showed presence of two main subclades (**Figure 1B**). The first subclade included eight genome sequences namely SP304 (ST285), VB35179 (ST1512), VB35435 (ST575), CIAT758 (ST10), PM194229 (ST10), PM194188 (ST10), PM2098 (ST10), and PM193665 (ST10) while the second subclade included rest of *A. baumannii* genomes. The phylogenetic analysis also showed that the *A. baumannii* genomes belonging to ST2 and ST622 formed two major monophyletic clades. The results of MLST and eBURST analysis when compared with the SNP phylogeny, showed that single and double locus variants were situated under same clades while the triple locus variants such as ST150 and ST374 were separated into different clades. The SNP data matrix showed variation between similar STs such as ST2, ST10, ST25 and ST1 (**Figure S1**). In case of ST622, nine (KSK1, KSK2, KSK6, KSK7, KSK10, KSK11, KSK18, KSK19, and KSK20) out of 10 genomes showed very low variation, with the only exception of VB16141 which showed higher variation. Overall, the phylogenetic analysis based on the alignment of concatenated SNP showed accordance with the STs detected in this study.

### Antibiotic resistance genes (ARGs)

**Figure 2:**
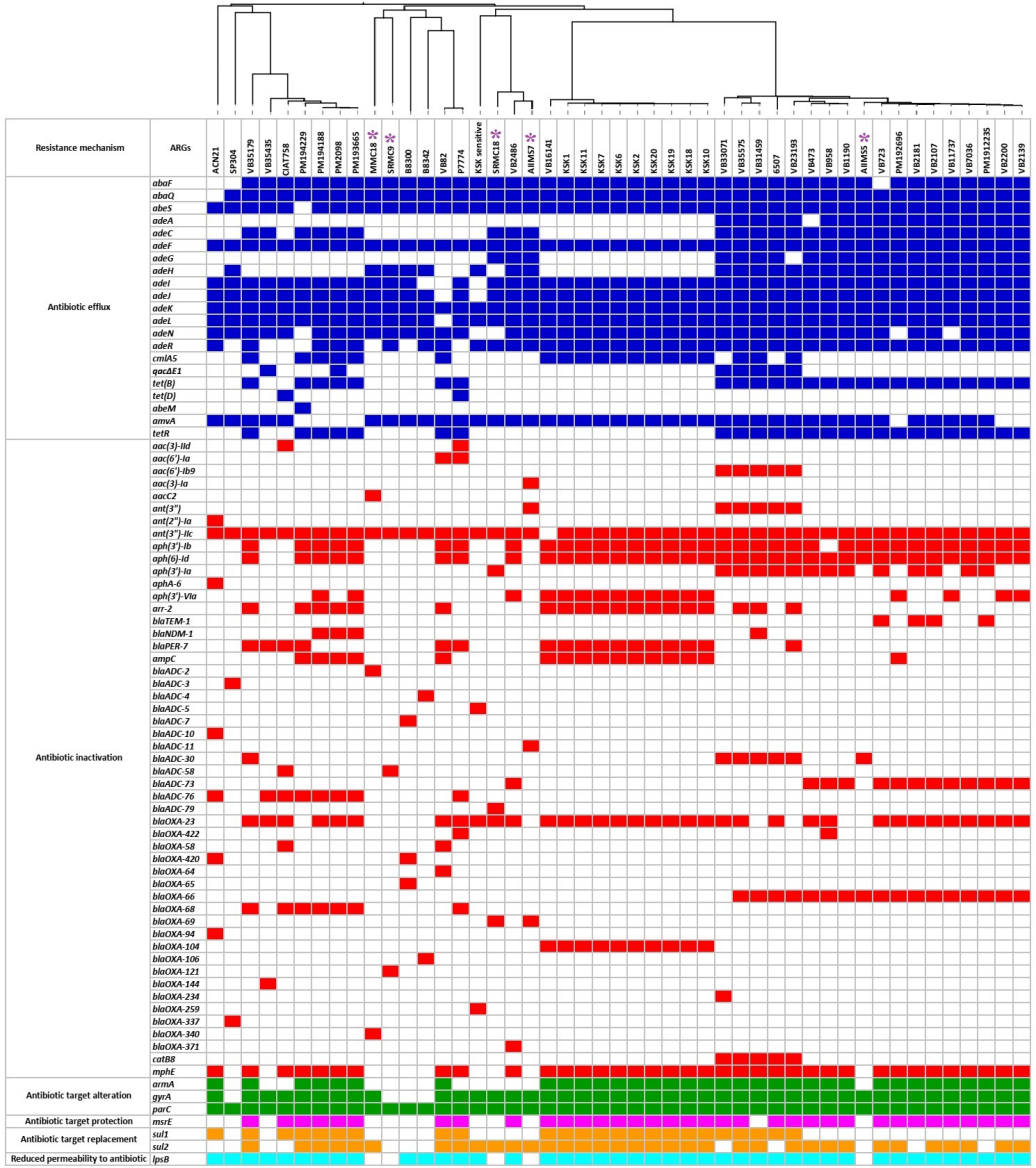
The predicted resistome of 47 A*. baumannii* genomes from India. The colored squares represent presence of ARGs while the empty or colorless squares represent absence of ARGs. The purple asterisks represent five *A. baumannii* isolates sequenced in this study.

**Figure 3:**
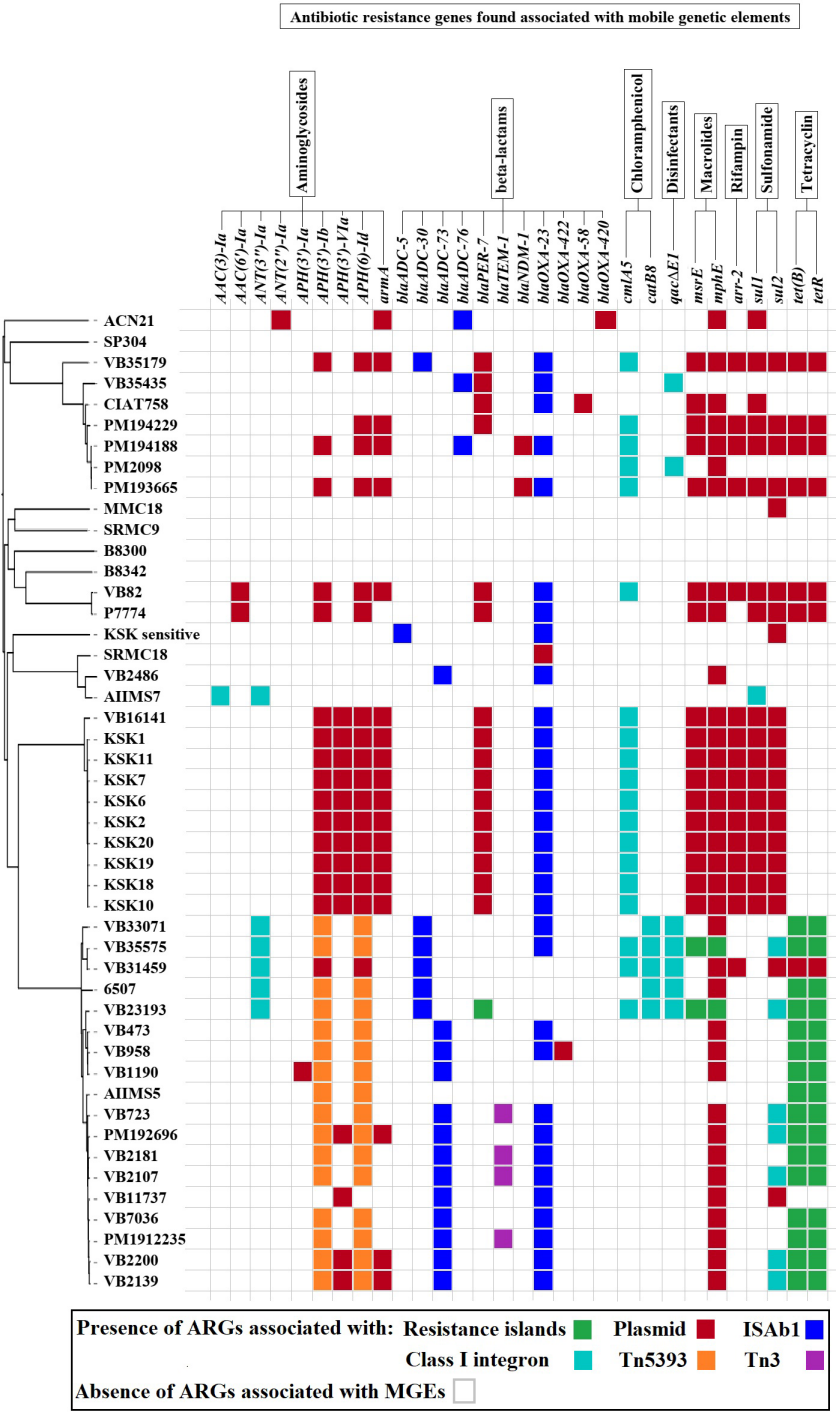
The acquired ARGs associated with MGEs predicted in the study.

The resistome analysis of 47 A*. baumannii* genomes showed presence of a total of 79 types of ARGs of intrinsic as well as acquired origin belonging to six resistance mechanisms, namely antibiotic efflux (n=21), antibiotic inactivation (n=51), antibiotic target alteration (n=3), antibiotic target protection (n=1), antibiotic target replacement (n=2), and reduced permeability to antibiotics (n=1) (**Figure 2**). All predicted β-lactamase genes showed 100% sequence similarity with the respective β-lactamase alleles present in the CARD database. Apart from the β-lactamase genes, the remaining ARGs showed 97 to 100% sequence similarity with the ARG sequences present in the CARD database. Three types of efflux pump associated ARGs, namely the resistance-nodulation cell division (RND) family, the major facilitator superfamily (MFS), and the small multidrug resistance (SMR) family were predicted in the study. We predicted 15 alleles of OXA-51 type and 12 alleles of ADC-type β-lactamases in this study. Additionally, OXA-23 and its allele OXA-422 as well as OXA-58 and its allele OXA-420 were predicted in the study. The most dominant type of family encoding β-lactam inactivating enzymes predicted was *blaOXA-23* (n=34/47), followed by blaADC-73 (n=13/47). Overall, 46 out of 47 A*. baumannii* genomes showed the coexistence of at least one type of the OXA-type-β-lactamases with one type of the ADC-type of cephalosporinases. The presence of putative genes for aminoglycoside acyltransferases (AAC family), aminoglycoside adenyltransferases (ANT family), and aminoglycoside phosphotransferases (APH family) was detected in 10, 46, and 38 genomes, respectively. The putative sulphonamide resistance conferring gene *sul*1 was predicted in 34 genomes, while *sul2* was detected in 42 genomes. The presence of *lpsB* gene associated with reduced permeability to antibiotics was predicted in 42 genomes. However, the genomes of AIIMS5, AIIMS7, MMC18, SRMC9, and SRMC18 did not show the presence of the *lpsB* gene. The quinolone resistance-determining region (QRDR) associated *parC* and *gyrA* were detected in 47 and 43 genomes, respectively. However, the presence of nonmutated *gyrA* observed in SRM9, SP304, B8342, and B8300 indicates a potential susceptibility of these isolates to quinolones. In case of acquired ARGs no significant difference was found between acquired ARGs predicted in ST2 as compared to the acquired ARGs predicted in rest of the 14 STs detected in the study (t = 0.882, p = 0.191, critical t = 1.756). Amongst the 18 A*. baumannii* isolates belonging to ST2, 14 (77%) showed the presence of *blaOXA-23*. A variation in the number of acquired ARGs was observed in the study. The genomes of SRMC9, SP304, B8300, and B8342 showed absence of acquired ARGs. A lower number of acquired ARGs was observed in AIIMS5 (n = 4), MMC18 (n = 1), and SRMC18 (n = 1) which were collected during 2005 in comparison to the remaining genomes which showed a relatively higher number (13 ≥ n ≥ 5) of acquired ARGs (**Figure 3**).

### Virulence factor genes (VFGs)

**Figure 4:**
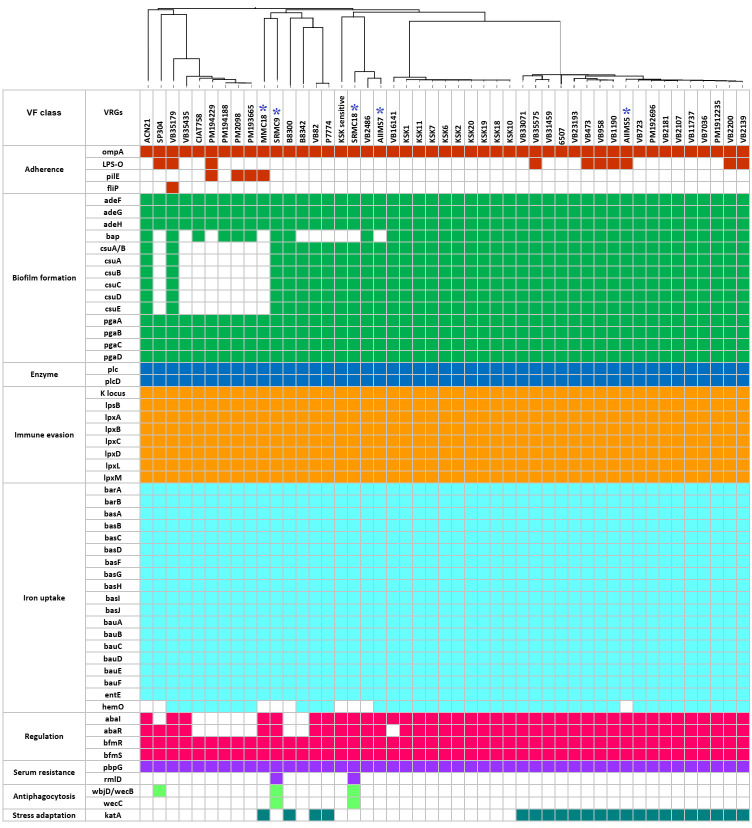
The predicted virulome of 47 A*. baumannii* genomes from India. The colored squares represent presence of VFGs while the empty or colorless squares represent absence of VFGs. The blue asterisks represent five *A. baumannii* sequenced in this study.

A total of 56 types of putative VFGs were predicted in 47 A*. baumannii* genomes (**Figure 4**). The predicted VFGs were grouped under nine categories namely, adherence (n=4), biofilm formation (n=14), enzyme (n=2), immune evasion (n=8), iron uptake (n=19), regulation (n=4), serum resistance (n=2), antiphagocytosis (n=2), and stress adaptation (n=1). The putative VFGs predicted in all 47 genomes included outer membrane protein A (*ompA*) involved in adherence and invasion, efflux pump *adeFGH* involved in biofilm synthesis, *pgaABCD* locus encoding poly-β-1-6-N-acetylglucosamine required for biofilm formation, phospholipase C and D, the capsular polysaccharide biosynthesis gene cluster K, the *lpx* genes involved in lipid biosynthesis, the two-component signal transduction system *BfmRS*, and penicillin binding protein G (*pbpG*). Except for the *basI* in VB35179, all putative genes associated with acinetobactin biosynthesis and intake (*barAB*, *basABCDFGHIJ*, *bauABCDEF*, and *entE*) were detected in all genomes. The presence of putative *bap* gene encoding biofilm associated protein, the putative *csuA/BABCDE* operon associated with pili synthesis and assembly, the putative *hemO* encoding the heme oxygenase, and the putative *abaI* inducer and its receptor *abaR* involved in the quorum sensing were predicted in 37, 39, 38, and 40 genomes respectively. A noticeable variation was observed in the putative genes encoding virulence factors such as O-antigen polysaccharide (*lpsO*), type IV pilin protein (*pilE*), flagellar biosynthesis protein (*flip*), enzyme involved in biosynthesis of dTDP-l-rhamnose (*rmlD*), catalase (*katA*), and the bifunctional enzyme UDP‐N‐acetylglucosamine 2‐epimerase/ManNAc kinase associated with antiphagocytic capsular polysaccharide synthesis (*wbjD/wecB*). Altogether, a vast variety of VFGs were predicted in this study.

### Mobile genetic elements (MGEs)

The plasmidSPAdes showed the presence of plasmid contigs in AIIMS5, AIIMS7, MMC18, and SRMC18. The CARD-based analysis of the detected plasmid contigs, as well as plasmids from other 42 A*. baumannii* genomes, predicted the presence of various putative ARGs associated with penems, aminoglycoside, tetracycline, macrolide, phenicol, rifamycin, sulfonamide, and heavy metal resistance (**Figure 3**).

Amongst the 274 prophage regions predicted, 108 were intact, 120 were incomplete and 46 were questionable. Further analysis classified predicted prophage regions into 5 families namely, siphoviridae (n=191), myoviridae (n=49), podoviridae (n=32), autographiviridae (n=1), and inoviridae (n=1) (**Figure S2**). The sequence showing similarity to PHAGE_Acinet_Bphi_B1251 was detected in all 47 genomes.

All 47 A*. baumannii* genomes analyzed in this study harbored numerous ISs of diverse bacterial origin. A total of 16 IS families, including Tn3, IS1, IS3, IS4, IS5, IS6, IS21, IS30, IS66, IS91, IS256, IS630, IS701, IS982, ISL3, and ISNCY, were detected in the study. We detected the presence of structures showing partial similarity to those of ISAba1-*blaADC* (n = 21/47) and ISAba1-*blaOXA-23* (n = 36/47).

All Tn3-type transposons detected in this study showed presence of antibiotics and heavy metal resistance genes. A total of four types of Tn3 similar to Tn5393, TnAs3, TnAs2, and Tn3 were predicted (**Figure S3**). Forty out of 47 genomes studied showed the presence of at least one type of Tn3 carrying at least one resistance gene. The Tn5393 detected in 35 genomes showed the presence of ARGs associated with aminoglycoside resistance (*strA*/*aph(3’’)-Ib*, *strB*/*aph(6)-Id*). In comparison, the TnAs3 detected in 29 genomes contained putative ARGs associated with an aminoglycoside, chloramphenicol, mercury, and quaternary ammonium compound resistance. The TnAs2, a truncated form of TnAs3, only showed presence of genes associated with mercury resistance. The structure of Tn3 showed presence of putative gene encoding β-lactamase-TEM-1b.

**Figure 5:**
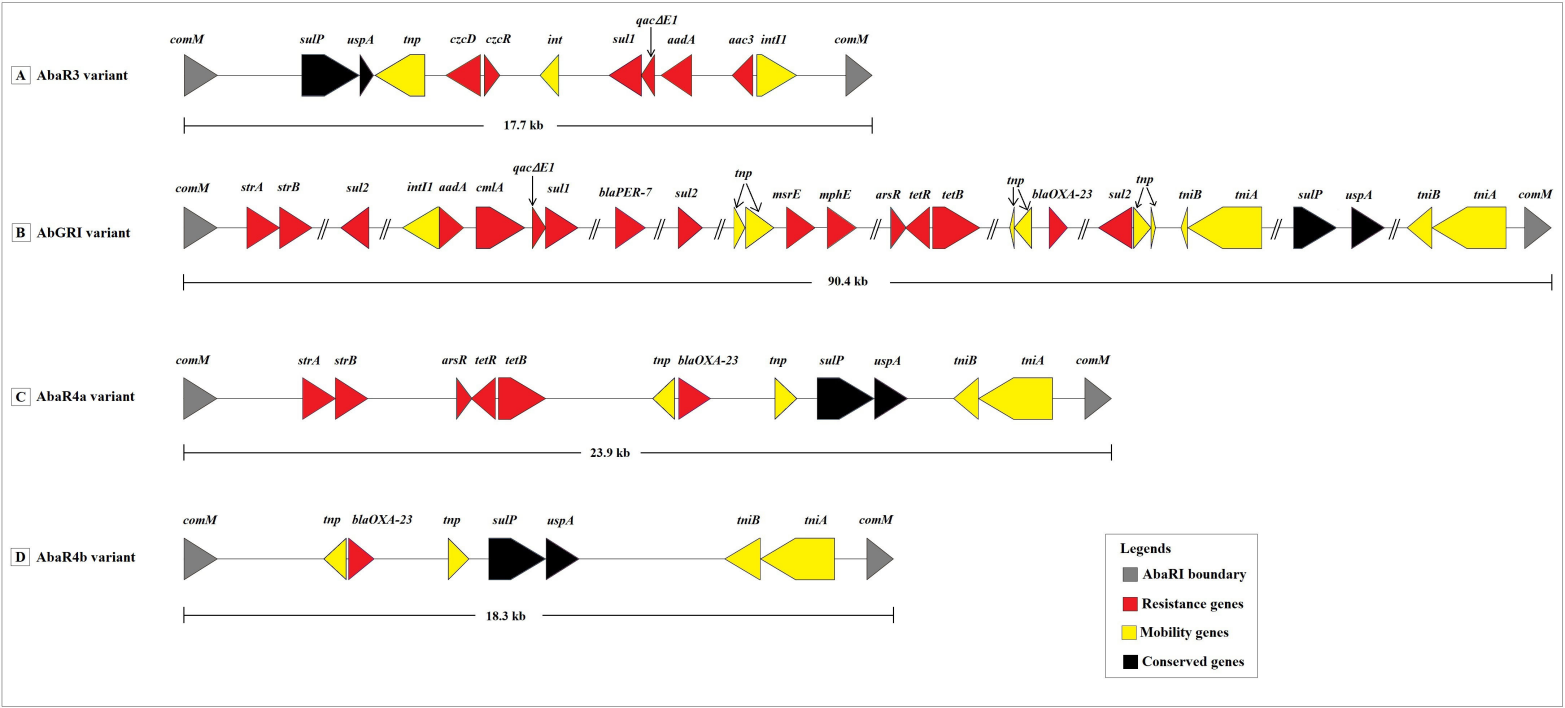
Schematic representation of four resistance island variants predicted in the study. **(A)** The AbaR3 variant predicted in AIIMS7 belonging to ST1. **(B)** The AbGRI variant predicted in VB23193 and VB35575 belonging to ST2. **(C)** The AbaR4a variant predicted in AIIMS5, VB33071, 6507, PM192696, PM1912235, VB473, VB723, VB958, VB1190, VB2107, VB2139, VB2181, VB2200, and VB7036 belonging to ST2. **(D)** The AbaR4b variant predicted in *A. baumannii* genomes belonging to ST10 (CIAT758, PM193665, PM194188), ST85 (VB82), ST575 (VB35435), ST25 (P7774), ST622 (VB16141, KSK6, KSK7, KSK10, KSK18, KSK19, KSK20), and ST1512 (VB35179). Legends: *comM*, ATPase; *sulP*, sulfate permease; *uspA*, universal stress protein A; *tnp*, Il-IS2 transposase; *czcD*, cobalt/zinc/cadmium resistance protein; *czcR*, Cd(II)/Pb(II)-responsive transcriptional regulator; *int*, prophage integrase; *sul1* and *sul2*, sulfonamide resistance protein; *qacΔE1*, quaternary ammonium compounds efflux transporter; *aadA*, aminoglycoside 3’’-nucleotidyltransferase; *aac3*, aminoglycoside N(3)-acetyltransferase; *intI1*, integron integrase; *tniB*, NTP binding protein; *tniA*, transposase; *strA*, aminoglycoside 3’’-phosphotransferase; *strB*, aminoglycoside 6-phosphotransferase; *arsR*, transcriptional regulator; *tetR*, tetracycline resistance regulatory protein; *tetB*, tetracycline resistance efflux protein; *cmlA*, chloramphenicol efflux pump; *blaPER-7*, class A β -lactamase; *msrE*, erythromycin resistance; *mphE*, macrolide 2’ phosphotransferase; *blaOXA-23*, class D β-lactamase.

The insertion of AbaRIs at *comM* gene was confirmed in 31 out of 47 genomes. The predicted AbaRIs varied in the length. The longest resistance island of size 90.4 kb was detected in VB23193 (ST2). In contrast, the shortest resistance island of size 17.7 kb was detected in AIIMS7 (ST1). Based on the sequence similarities shown with previously known AbaRIs sequences, we classify predicted AbaRIs into four types namely, ABaR3 variant, AbGRI variant, AbaR4a variant, and AbaR4b variant (**Figure 5**). The presence of truncated segment of Tn6022 carrying genes such as transposase (*tniA*), NTP-binding protein (*tniB*), universal stress protein (*uspA*), and sulfate permease (*sulp*) was found in all resistance islands except for the ABaR3 variant found in AIIMS7 which was a ST1 isolate (**Figure 5A**). The ABaR3 variant detected in ST1 (AIIMS7) possessed putative genes involved in cadmium and zinc resistance (*czcD, czcR*). However, it lacked the *blaOXA-23* which was found in 27 out of 31 AbaRIs detected in this study. The ABaR3 variant predicted in AIIMS7 also showed presence of a class I integron gene cassette carrying putative genes conferring resistance to disinfectant (*qacEΔ1*), sulphonamide (*sul1*), and aminoglycoside (*aadA*, *aac3*). The two types of resistance islands were observed in ST2 genomes namely AbGRI variant and AbaR4a variant (**Figures 5B, C**). The complex AbGRI variants detected in VB23193 (ST2) and VB35575 (ST2) were longer in length as compared to the AbaR4a variant observed in the remaining ST2 genomes. Despite the complex nature and length variation in both ST2 variants (AbGRI and AbaR4) showed presence of certain common genes such as tetracycline resistance genes (*tetB*, *tetR*) as well as the truncated fragment of Tn5393 carrying *strA* and *strB* genes. Another difference we observed in AbaRI variants detected in ST2 genomes was, the presence of chloramphenicol (*cmlA*) and macrolides resistance (*mphE*, *msrE*) genes in AbGRI variant while their absence in AbaR4 variant. A fourth type of AbaRI variant namely AbaR4b was predicted in *A. baumannii* genomes belonging to ST10, ST85, ST575, ST25, ST622, and ST1512 (**Figure 5D**). Similar to AbGRI variant and AbaR4a variant predicted in ST2 isolates the AbaR4b showed presence of *blaOXA-23*. However, it lacked *tetB*, *tetR* and *asrR* genes. Overall, the comparative analysis of 47 A*. baumannii* genomes showed presence of multiple types of MGEs carrying putative resistance genes associated with antibiotics, disinfectants, and heavy metal resistance (**Figure 3**).

## Discussion

In India, the majority of the studies concerning *A. baumannii* focus on the phenotypic, genotypic or epidemiological characterization (Saranathan et al., 2014; Vijayakumar et al., 2016). The antimicrobial surveillance studies based on phenotypic characterization show that *Acinetobacter* spp. is the second most isolated pathogen (45%) after *Pseudomonas* spp. (52%). This report also emphasizes that>70% *A. baumannii* isolates show non-susceptibility to most antibiotics tested except colistin (Walia et al., 2019). The genotyping methods such as PCR-based oligotyping generally remain limited to molecular characterization of one or more genetic determinants associated with resistance or virulence (Kumar et al., 2010; Upadhyay et al., 2018). Despite the usefulness of routine phenotypic or molecular typing methods, the NGS provides an extraordinary insight into the genomic organization of outbreak causing pathogens. The data generated through genome sequencing can be further used to track clinically important pathogens like *A. baumannii* circulating through the Indian population. However, only a handful of studies based on these cutting-edge techniques exploring the dynamic genome organization of *A. baumannii* are reported from India (Kumar et al., 2015; Vijayakumar et al., 2019). Considering this scenario, we have performed a comprehensive comparative genome analysis of 47 clinical *A. baumannii* isolates collected between 2005 and 2020 from India.

The worldwide spread of carbapenem resistance *via A. baumannii* strains belonging to IC2 clonal lineage is a well-known factor (Gogou et al., 2011; Hamidian and Nigro, 2019). A study conducted by Kumar et al. has confirmed the presence of IC2 clones of *A. baumannii* carrying *blaOXA-23* like carbapenamases in India (Kumar et al., 2019). Consistent with this finding, a similar trend confirming the presence of larger number (n=18, 38.3%) of *A. baumannii* isolates belonging to ST2 genotype (IC2 lineage) was observed in this study. The ST2 strains in our study have shown presence of significantly higher number of ARGs as compared to the other predicted STs. The presence of *blaOXA-23* in almost 77% of ST2 *A. baumannii* genomes indicates their contribution in expansion of carbapenem resistance in Indian scenario. Apart from the ST2, the ST622 and ST10 were other major sequence types included in the current study. The occurrence of ST622, a relatively novel sequence type has been reported previously from Nepal while the ST10, a sequence type of a hypervirulent *A. baumannii* was reported from Iran and China (Shrestha et al., 2015; Abhari et al., 2019). Moreover, STs 622 and 10 have shown presence *blaOXA-23* suggesting their potential role in spread of carbapenem resistant *A. baumannii* clones in India.

Gene content variation within *A. baumannii* genomes was reported previously (Sahl et al., 2015; Wallace et al., 2016). As per our study, a variation in the occurrence of ARGs was found between the genomes compared. The SRMC9, B8342, B8300 and SP304 genomes showed presence of a smaller number of ARGs than that predicted in the rest of the genomes included in the study. The combined action of intrinsic and acquired resistance in *A. baumannii* is considered as a serious barrier in the treatment of *A. baumannii* infections (Hood et al., 2013; Yoon et al., 2015; Juan et al., 2017; Jarlier et al., 2019). Our analysis has shown presence of ARGs of both intrinsic (*AdeIJK*, *blaOXA-51* like β-lactamases, *AmpC* cephalosporinase, *gyrA*, *parC*) as well as of acquired (*blaOXA-23 type, blaOXA-58 type, aac, aph, ant, cml, cat, armA, msrE, qacΔE, sul, tet*) origin which signifies the clinical importance of *A. baumannii*. Overall, the versatile resistome predicted in this study highlights a widespread of MDR *A. baumannii* capable of resisting β-lactams, cephalosporins, carbapenems, aminoglycosides, and quinolones in India.

The pathogenicity illustrated by *A. baumannii* is a concerted action of several virulence factors together (Moubareck and Halat, 2020). The VFs that ensure survival and persistence of *A. baumannii* under diverse environmental conditions are mainly associated with motility, adherence, and biofilm formation (Sarshar et al., 2021). The putative VFs predicted in this study such as surface associated virulence factors such as *OmpA*, *Bap*, *Csu* pili, and *poly-β-(1–6)-N-acetyl glucosamine* (PNAG) which are known for their active role in motility, adherence and biofilm formation (Harding et al., 2018). The VFGs associated with synthesis of LPS, capsular polysaccharides, phospholipase, acinetobactin and heme oxidase predicted in our study were previously accounted for exhibition of serum resistance, immune invasion and iron acquisition capacity of *A. baumannii* (Sanchez-Larrayoz et al., 2017). All 47 isolates possessed an almost equal number of VFGs. In this study we did not find any linkage between VFGs and MGEs. However, considering the dynamic nature of MGEs, the possibility of acquisition of VFGs and their subsequent spread cannot be overruled. Overall, the combination of VFGs and ARGs predicted in this study provides ample evidence to suggesting the potential of *A. baumannii* to survive using ‘persist and resist’ strategy.

*A. baumannii* possesses an ‘open’ pan-genome which describes its ability to acquire new genes *via* various MGEs (Imperi et al., 2011) (**Figure S4**). The presence of numerous MGEs such as plasmids, insertion sequences, transposons, and resistance islands explain the high genome plasticity observed in this bacterium (Dziri et al., 2018; Cerezales et al., 2020). Plasmids are one of the most studied MGEs in *A. baumannii*. They have been shown to carry ARGs associated with carbapenems, aminoglycosides, tetracyclines, quinolones, macrolides, sulfonamides, and polymyxins resistance (Brovedan et al., 2020). In the present study, we have predicted the presence of plasmids carrying similar types of ARGs.

Apart from the plasmids, the role of insertion sequences, transposons, resistance islands, and prophages in conferring antimicrobial resistance in *A. baumannii* has been well documented (Pagano et al., 2016; Partridge et al., 2018) The increasing adaptability and prevalence of *A. baumannii* strains belonging to the ST2/IC2 genotype is generally linked to its ability to acquire and spread resistance determinants through activity of MGEs (Zarrilli et al., 2013). The prevalence of *A. baumannii* clones containing variants of AbaRI have also been reported (Li et al., 2015). One of the characteristic feature of resistance islands is presence of transposons similar to Tn5393 and Tn2006 which are generally found associated with carriage of aminoglycoside (*strA*, *strB*) and β-lactam (*blaOXA-23*) resistance genes (Zhou et al., 2011; Nigro and Hall, 2015; Lee et al., 2016). Amongst the four types of AbaRIs variants predicted in this study, two variants showed presence of Tn5393 linked *strA*, *strB* while three variants showed presence of Tn2006 associated *blaOXA-23* gene. Another reason for the carbapenem resistance exhibited by *A. baumannii* isolates is overexpression of *blaOXA-23* gene due to the insertion of ISAba1 element (Viana et al., 2016). The current study predicted the presence of ISAba1 linked *blaOXA-23* suggesting the possibility of *A. baumannii* analyzed in this study to exhibit an increased resistance to carbapenems. Similarly, the ISAba1-*blaADC* linkage was observed to play a significant role in cephalosporin resistance (Lopes and Amyes, 2012; Biglari et al., 2015). The ISAba1-*blaADC* linkage predicted in this study using in silico approach represents the potential of these isolates to confer cephalosporin resistance. The association between presence of class 1 integron cassettes and antibiotic resistance in *A. baumannii* was reported previously (Huang et al., 2015). The findings in this study showing presence of class 1 integron cassettes in association with resistance islands points towards the dynamic nature of *A. baumannii* genomes.

The role of bacterial prophages in transfer of ARGs have been reported in several studies (Chene et al., 2006; Haaber et al., 2016). In the present study, we have observed presence of multiple copies of prophages in all 47 genomes. However, no ARGs were detected within these prophage regions. Nevertheless, a further study to analyze potential of these prophage regions in transfer of ARGs is needed. In summary, the presence of plasmids, Tn3 transposons, AbaRIs associated with putative genes conferring resistance to β-lactams, aminoglycosides, chloramphenicol, quaternary ammonium compounds, and heavy metals was predicted in this study. In addition to these findings, we also observed the presence of higher number of ARGs associated with MGEs in *A. baumannii* isolates collected after 2014 as compared to the isolates collected in 2005 (**Figure 3**).

The inclusion of a fewer number of complete *A. baumannii* genomes from India could be considered one of the limitations of this study. Additionally, the genomes analyzed in this study only represent the north and south region of the country. The analysis does not depict a wide distribution of *A. baumannii* isolates throughout India and therefore provides a partial genomic diversity. Furthermore, the study limits itself to *A. baumannii* genomes collected from 2005 and from 2014 to 2020. The study would have been more decisive if the genomes from a wide temporal and geographical region were available.

In conclusion, this study provides a detailed account of genetic determinants associated with multidrug resistance and virulence in clinically significant *A. baumannii* isolates from India. Despite the diversity in sequence types, a dominance of *A. baumannii* isolates associated with ST2 was observed. Furthermore, the study showed a prevalence of diverse MGEs in *A. baumannii* genomes which highlights its ability to adapt and evolve. The study also suggests a need for the real time functional analysis of MGEs involved in dissemination of multidrug resistance. Considering this pathogen’s challenging nature, we strongly recommend the use of NGS in monitoring and thereby controlling the spread of outbreak causing *A. baumannii* isolates in India.

## Data availability statement

The datasets presented in this study can be found in online repositories. The names of the repository/repositories and accession number(s) can be found in the article/**Supplementary Material**.

## Author contributions

SK conceived and designed the study. SK wrote the article, generated all tables and figures in the article. NC and DD helped with the initial quality check of the sequencing data for five *A. baumannii* genomes. Further, NC and DD helped with interpretation of results obtained through in silico analysis. EK helped with the statistical analysis. KP helped with manuscript reviewing and editing. All authors contributed to the article and approved the submitted version.

## Funding

This work was supported by the Department of Science and Technology, India under the scheme Promotion of University Research and Scientific Excellence (DST-PURSE, GOIA670), University with Potential for Excellence Phase II program (UGC-262-A-2) implemented at Savitribai Phule Pune University, and the Director, NCCS, Pune. SK is a recipient of the CMSRF 2019 fellowship provided by SARTHI, Pune. The funders had no role in study design, data collection and analysis, decision to publish, or preparation of the manuscript. The authors apologize for this error and state that this does not change the scientific conclusions of the article in any way. The original article has been updated.

## Acknowledgments

We thank the bioinformatics team at the National Centre for Microbial Resource (NCMR), Pune, for their technical assistance. This work was supported by the Department of Microbiology, Savitribai Phule Pune University. The funding for bacterial genome sequencing was procured from DST-PURSE (GOI-A-670) implemented at Savitribai Phule Pune University.

## Conflict of interest

The authors declare that the research was conducted in the absence of any commercial or financial relationships that could be construed as a potential conflict of interest.

## Publisher’s note

All claims expressed in this article are solely those of the authors and do not necessarily represent those of their affiliated organizations, or those of the publisher, the editors and the reviewers. Any product that may be evaluated in this article, or claim that may be made by its manufacturer, is not guaranteed or endorsed by the publisher.
