# Corrigendum: Diversity of resistant determinants, virulence factors, and mobile genetic elements in *Acinetobacter baumannii* from India: A comprehensive in silico genome analysis

**DOI:** 10.3389/fcimb.2022.1130394

**Published:** 2023-01-10

**Authors:** Shital N. Kumkar, Ekta E. Kamble, Nikeeta S. Chavan, Dhiraj P. Dhotre, Karishma R. Pardesi

**Affiliations:** ^1^ Department of Microbiology, Savitribai Phule Pune University, Pune, Maharashtra State, India; ^2^ National Centre for Cell Science, Savitribai Phule Pune University Pune, Maharashtra State, India

**Keywords:** antibiotic resistance genes (ARGs), multidrug resistance (MDR), virulence factor genes (VFGs), mobile genetic elements (MGEs), India, *Acinetobacter baumannii*

In the published article, there was an error in the author list, and author Dr. Dhiraj P. Dhotre was erroneously excluded as the corresponding author. The corrected author list appears below.

“Shital N. Kumkar^1^, Ekta E. Kamble^1^, Nikeeta S. Chavan^2^, Dhiraj P. Dhotre^2^*, Karishma R. Pardesi^1^*”

In the published article, there was an error in the Funding statement. We did not acknowledge the Director, NCCS, Pune for providing financial support for article processing charges. The correct Funding statement appears below.

