# Supplementary material for: Diversity of resistant determinants, virulence factors, and mobile genetic elements in Acinetobacter baumannii from India: A comprehensive in silico genome analysis
Source: Front Cell Infect Microbiol. 2022 Nov 28;12:997897. doi: 10.3389/fcimb.2022.997897 (PMC9742364; doi:10.3389/fcimb.2022.997897)
Supplement: Supplementary file 1 [file DataSheet_1.docx]

Supplementary Material

Diversity of resistant determinants, virulence factors and mobile genetic elements in *Acinetobacter baumannii* from India: A comprehensive in silico genome analysis

## Supplementary Figures

**Figure S1**

**
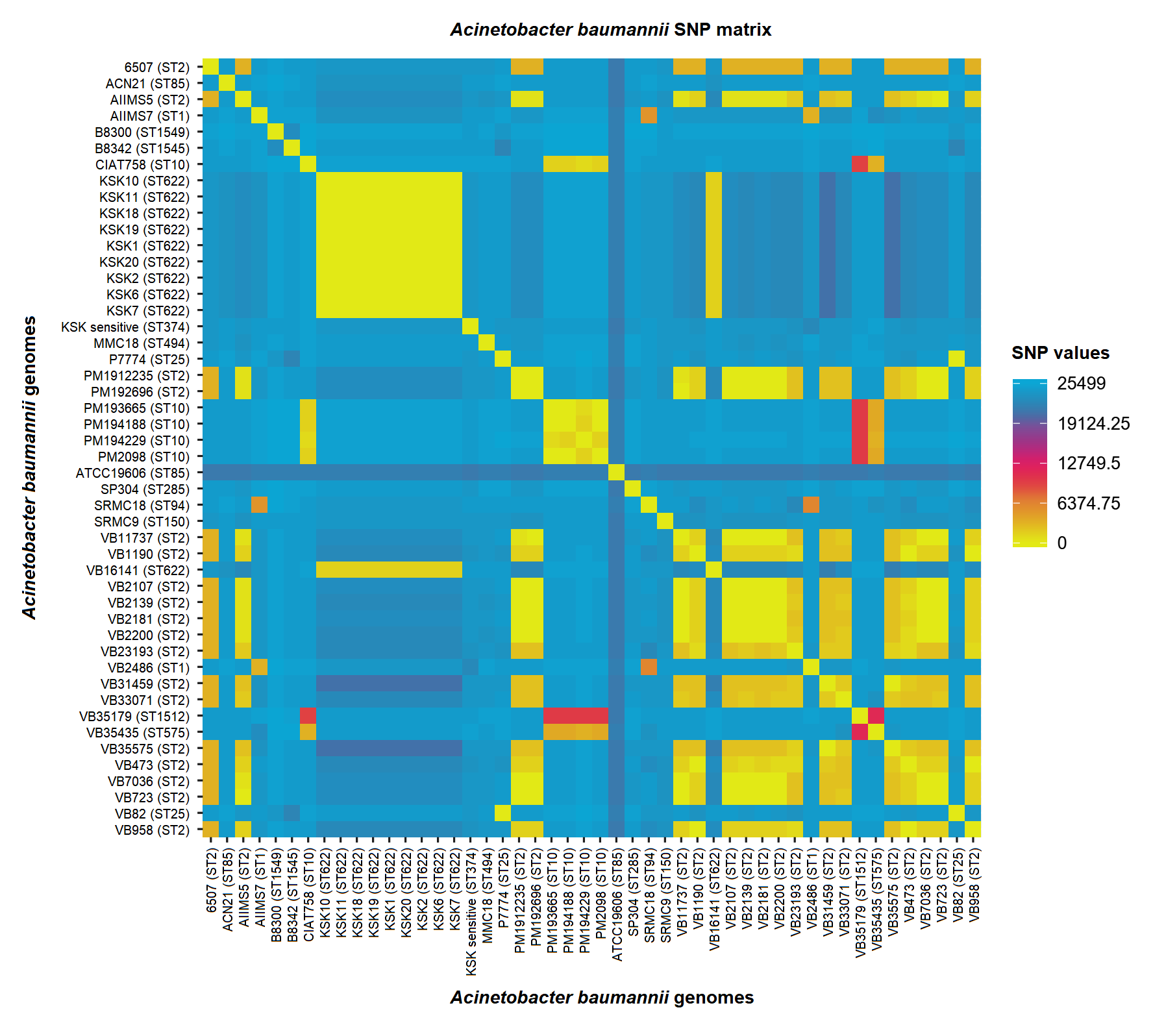
**

**Figure S1 |** The SNP matrix showing single nucleotide variations in 47 *A. baumannii* genomes compared in the study


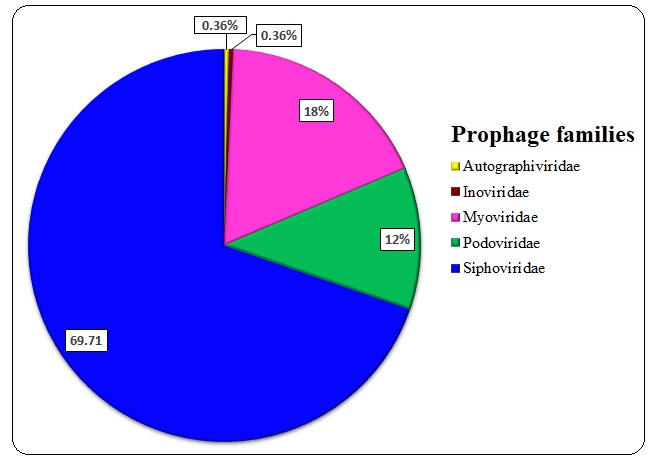
**Figure S2**

**Figure S2 | Percent distribution of prophage families predicted in 47 *A. baumannii* genomes from India.**


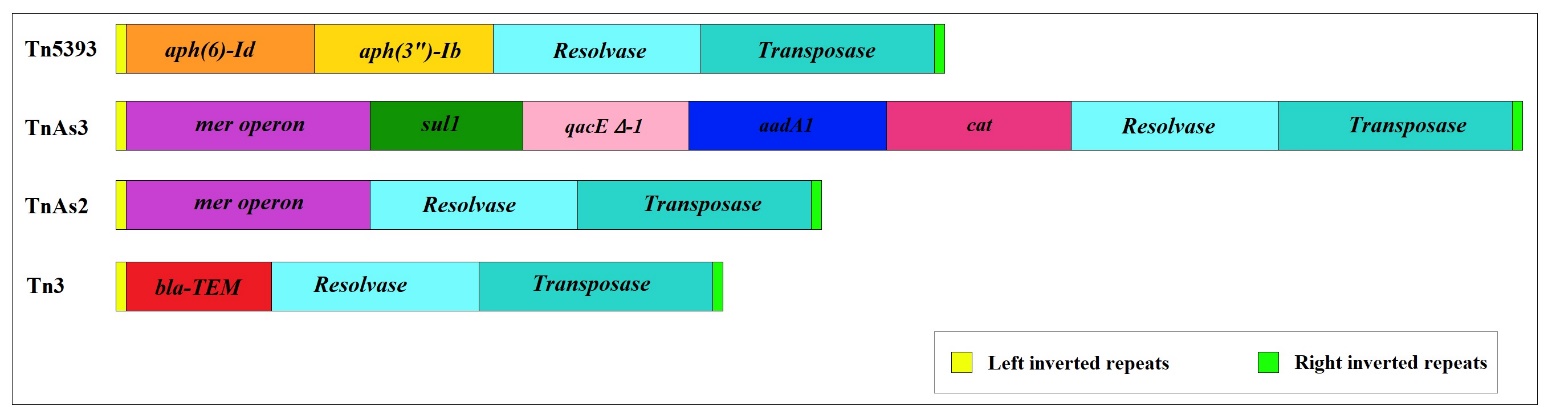
**Figure S3**

**Figure S3 | Schematic representation of types of Tn3 transposons predicted in the study.** Legends: *aph(6)-Id*, aminoglycoside 6-phosphotransferase; *aph(3”)-Ib*, aminoglycoside 3''-phosphotransferase; *mer*, mercury resistance; *sul1*, sulfonamide resistance protein; *qac∆E1*, quaternary ammonium compounds efflux transporter; *aad1*, aminoglycoside nucleotidyltransferase ; *cat*, chloramphenicol acyltransferase; *bla-TEM*, TEM β-lactamase

**Figure S4**


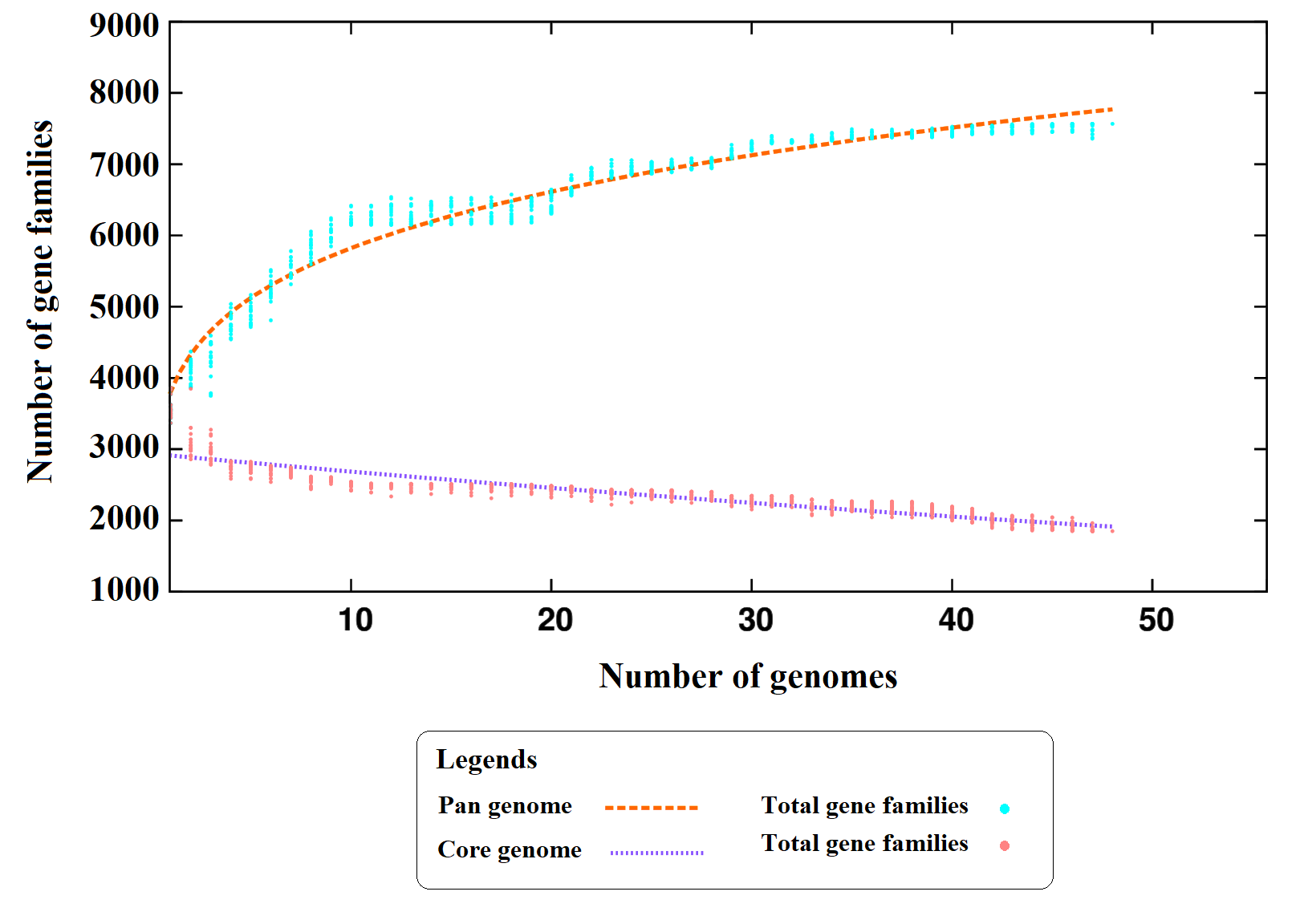


**Figure S4 |** Core and pan genome analysis of 47 *A. baumannii* genomes using Bacterial Pan Genome Analysis (BPGA) pipeline. The continuous increase in the pan genome curve shows that *A. baumannii* possesses an open pan genome while its core genome appears relatively stable.

**Supplementary Table S1**

| ***A. baumannii* isolates** | **Number of Reads** | **Average read length (bp)** | **No. of contigs** | **Contigs N50** |
| --- | --- | --- | --- | --- |
| AIIMS5 | 5117234 | 150 | 71 | 144127 |
| AIIMS7 | 4202400 | 150 | 95 | 286824 |
| MMC18 | 4870332 | 150 | 114 | 95232 |
| SRMC9 | 5379421 | 150 | 33 | 388048 |
| SRMC18 | 6410407 | 150 | 104 | 87542 |

**Table S1** Assembly statistics for five *A. baumannii* isolates sequenced in this study

**Data Availability Statement**

The genome sequences of five A. baumannii namely AIIMS5, AIIMS7, MMC18, SRMC9, and SRMC18 reported in the current study have been deposited at GenBank under accession number JAKZFE000000000, JAKZFF000000000, JAKZFG000000000, JAKZFH000000000, and JAKZFI000000000 respectively. The accession number of remaining 42 A. baumannii genomes used in this study for comparison are mentioned in Table 1.
